# Differential Consumption of Four Aphid Species by Four Lady Beetle Species

**DOI:** 10.1673/031.010.3101

**Published:** 2010-04-06

**Authors:** Christy Finlayson, Andrei Alyokhin, Serena Gross, Erin Porter

**Affiliations:** The University of Maine School of Biology and Ecology, 5722 Deering Hall, Room 202, Orono, ME 04469-5722

**Keywords:** predation, biological control, competition, non-native species

## Abstract

The acceptability of four different aphid species *Macrosiphum albifrons* (Essig), *Macrosiphum euphorbiae* (Thomas), *Macrosiphum pseudorosae* Patch, and *Myzus persicae* (Sulzer) (Hemiptera: Aphididae), as prey for four lady beetle species, one native species *Coccinella trifasciata* L, and three non-native *Coccinella septempunctata* L, *Harmonia axyridis* Pallas, *Propylea quatuordecimpunctata* L (Coleoptera: Coccinellidae) were tested in the laboratory. The relative field abundance of adults of the same lady beetle species on host vegetation, *Lupinus polyphyllus* Lindley (Fabales: Fabaceae), *Solanum tuberosum* L (Solanales: Solanaceae), and *Rosa multiflora* Thunberg (Rosales: Rosaceae), both with and without aphids present was also observed. In the laboratory, *H. axyridis* generally consumed the most aphids, while *P. quatuordecimpunctata* consumed the fewest. The exception was *P. quatuordecimpunctata*, which consumed a greater number of *M. albifrons* nymphs, and *C. trifasciata*, which consumed a greater number of *M. albifrons* nymphs and adults, compared with the other two beetle species. Lady beetles consumed fewer *M. albifrons* compared with the other three aphid species, likely because of deterrent compounds sequestered by this species from its host plant. In the field, *P. quatuordecimpunctata* was the most abundant species found on *L. polyphyllus* and *S. tuberosum*.

## Introduction

Lady beetles (Coleoptera: Coccinellidae) are known to be voracious predators of plant pests such as aphids (Hemiptera: Aphididae) (Hodek 1973; Gordon 1985). It is often assumed that aphidophagous lady beetles are highly polyphagous, consuming most (if not all) aphid species that they encounter (Pedigo and Rice 2006). However, there is evidence that not every aphid species is equally suitable for every lady beetle species (Obrycki and Orr 1990; Phoofolo and Obrycki 1997; Kalushkov 1998; Michaud 2000; Kalushkov and Hodek 2004; Mignault et al. 2006). For example, Michaud (2000) conducted choice tests with seven lady beetle species and two aphid species, *Toxoptera citricida* and *Aphis spiraecola*. Although all lady beetles tested consumed both aphid species, four species *Coccinella septempunctata*, *Coleomegilla maculata fuscilabris*, *Coelophora inaequalis*, and *Olla v-nigrum*, were not able to complete their developmental cycle with either aphid species. Depending on the aphid species consumed and the addition of supplements (pollen) to the diet, the other three species, *Hippodamia convergens*, *Cycloneda sanguinea*, and *Harmonia axyridis*, varied considerably in the number of eggs laid, egg viability, larval development time, and adult weight.

Lady beetles are commonly released to combat a diverse range of pests (Gordon 1985; Koch 2003), despite the fact that little is known about specific prey preferences of different species. The success of such pest control measures depends, in part, upon the willingness of the lady beetles to consume the pest in question. Releases of non-native species may supplement pest control by native species when their prey species do not overlap or when prey is plentiful. Native lady beetle abundance, however, may be reduced through competition with non-native species with overlapping prey preferences. Additionally, non-native lady beetles may alter aphid community structure. Determining differences in prey consumption by different lady beetle species may provide insight into changes that occur in systems where non-native species become established. In the laboratory, one native and three non-native lady beetle species were provided four different species of aphid prey and their consumption was recorded. To determine if any differences documented in the laboratory were reflected in the field, lady beetle species were observed for their association with these aphids under field conditions.

## Materials and Methods

### Study species

The four lady beetle species chosen for this study are aphidophagous (Gordon 1985) and abundant in Maine in the same habitats (Finlayson et al. 2008). The native lady beetle species used was *Coccinella trifasciata perplexa* Mulsant, which is found from Labrador south to New Jersey and west to California and Alaska (Gordon 1985). The non-native lady beetle species used were *Coccinella septempunctata* L., *Harmonia axyridis* Pallas, and *Propylea quatuordecimpunctata* L. These three species are Palearctic in origin and were intentionally and inadvertently introduced in North America. *C. septempunctata* has been established in North America since 1973 (Angalet and Jacques 1975), *H. axyridis* since 1988 (Chapin and Brou 1991; Tedders and Schaefer 1994), and *P. quatuordecimpunctata* since 1968 (Wheeler 1990).

Four aphid species that are abundant and readily available in the region were chosen to serve as the prey for the selected lady beetle species. The potato aphid, *Macrosiphum euphorbiae* (Thomas), feeds on over 200 plant species (Blackman and Eastop 1984). The green peach aphid, *Myzus persicae* (Sulzer), feeds on over 40 different plant families (Blackman and Eastop 1984). The hosts of the rose aphid, *Macrosiphum pseudorosae* (Patch), include the genus *Rosa* and a variety of herbaceous plants (Foottit and Maw 1997). The lupine aphid, *Macrosiphum albifrons* Essig, is a specialist, feeding exclusively on plants in the genus *Lupinus* (Blackman and Eastop 1984). While *M. persicae* is believed to be Palearctic in origin (Blackman and Eastop 1984), the other three aphid species are Nearctic (Stroyan 1981; Blackman and Eastop 1984).

### Laboratory trials

Lady beetles were collected from the field 48–72 hours before test initiation, maintained on a 50/50 diet of honey/egg yolk, then provided with water, but no food, for 48 hours before test initiation. Lady beetles were collected from a variety of locations and plants in Orono, Maine (44.8835° N, 68.6721° W), that included mixed shrub (*Solidago* sp., *Rubus* sp., *Prunus* sp., *Rosa* sp., *Cornus sericea*, *Alnus* sp.), apple (*Malus* sp.), grain (*Hordeum* sp., *Avena* sp.), mixed organic crops (*Solanum lycopersicon*, *Allium* sp., *Brassica* sp., *Pisum* sp., *Phaseolus* sp.) and fallow fields (*Phleum pratense*, *Trifolium* sp., *Cirsium* sp., *Vicia* sp., *Fragaria* sp.).

Potato aphids and green peach aphids were obtained from colonies maintained in the laboratory. The colonies were originally founded by aphids collected from potato, *Solanum tuberosum* (Solanales: Solanaceae), in Presque Isle, Maine, and then maintained for at least 20 generations on excised potato foliage in the laboratory. Rose and lupine aphids were collected in the field from host vegetation including multi-flora rose, *Rosa multiflora* Thunberg (Rosales: Rosaceae), and lupine, *Lupinus polyphyllus* Lindley (Fabales: Fabaceae), respectively, and then maintained in the laboratory on excised host vegetation for up to 3 days before use in trials.

For each experiment, 10 aphids of the same species were placed, using a paintbrush, on an excised leaflet held within a 100 × 15 mm polystyrene Petri dish. Leaves used in trials were from the host plants from which aphids were collected in the field, as previously stated. Each trial was initiated when a single lady beetle previously housed in a separate Petri dish was added to the Petri dish containing the aphids by quickly exchanging lids between the two Petri dishes when the lady beetle was on the lid. After 24 hours, the beetle was removed and the number of aphids remaining in the dish was recorded. When a partial aphid remained, it was estimated to the 0.25 aphid. The experiment was conducted separately with adult apterae and with first to second instars. Sixty trials were conducted with each lady beetle species/aphid species pairing: 30 replicates with adult aphids and 30 replicates with the nymphs.

Lady beetles, aphid colonies, and test dishes were housed in Percival I-33VL Intellus environmental chambers at a 16:8 L:D photoperiod and 20° C. Trials with *M. euphorbiae* and *M. albifrons* were conducted in 2005, from June 16 to August 12 and from June 2 to August 12, respectively. Trials with *M. persicae* and *M. pseudorosae* were conducted in 2006, from May 24 to August 16 and from August 10 to August 24, respectively. Trials were conducted continuously throughout the range of dates and in random order with respect to beetle species, aphid species, and choice of aphid nymph or adult.

### Field observations

Plots of *L. polyphyllus*, *S. tuberosum*, and *R. multiflora* were observed for 30 minutes each in Orono, ME (44.8974°N, 68.6873°W). Observations were made between 10:00 am and 2:00 pm in plots at least 0.1 ha in size where the vegetation of interest was dominant (≥ 50%). The number of adult lady beetles on host vegetation where aphids were absent (designated “absent”) or where *M. albifrons*, *M. euphorbiae*, or *M. pseudorosae* were present (designated “present”) was recorded. Because aphid populations were fairly contiguous where present, with no break in distribution greater than approximately one meter, each of the plots observed was designated as either “absent” or “present.” Forty observation trials were conducted for each of the three species. *M. persicae* were not found in the field in numbers sufficient to conduct observations. *M. albifrons* colonies were observed from June 2 to July 12, 2005, *M. euphorbiae* colonies were observed from June 17 to July 30, 2005, and *M. pseudorosae* colonies were observed from June 20 to August 24, 2006.

### Statistical analyses

Normality of laboratory-generated data was tested using the Wilk-Shapiro test (PROC UNIVARIATE; SAS Institute Inc. 2002). The data were transformed using rank transformations (Conover and Iman 1989). Means and standard errors reported in this paper were calculated from the untransformed data. Differences between lady beetle species were analyzed separately for each aphid species using one-way ANOVA followed by Tukey's multiple comparison test (PROC GLM; SAS Institute Inc. 2002). Analyses were conducted separately for aphid nymphs and adults.

Poisson regression (PROC GENMOD; SAS Institute Inc. 2002; SAS Institute Inc. 2005) was used to analyze lady beetle count data generated during field observations. Each plant species observed was analyzed separately, with the number of lady beetles as the response variable and lady beetle species and aphid presence/absence as the predictor variables. Overdispersion for *M. albifrons* and *M. pseudorosae* was corrected using a multiplicative overdispersion factor (Pearson chi-square divided by degrees of freedom) (Cox 1983; Allison 1999; SAS Institute Inc. 2005).

## Results

### Laboratory trials

**Figure 1.**
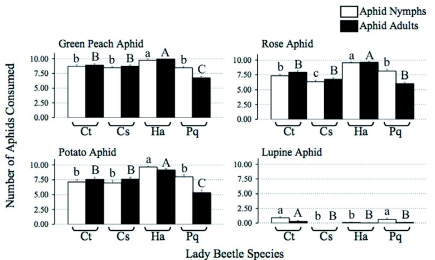
Mean (± standard error) consumption of aphids by different lady beetle species (Ct = *Coccinella trifasciata*, Cs = *Coccinella septemmpunctata*, Ha = *Harmonia axyridis*, Pq = *Propylea quaturodecimpunctata*). For each aphid species, nymphs and adults were analyzed separately; means with the same letter are not significantly different. High quality figures are available online.

There were always significant differences in the numbers of aphids consumed by different lady beetle species (Figure 1). *H. axyridis* consumed the most nymphs and adults of *M. persicae* (nymphs: F_3,116_ = 6.27, p < 0.0006; adults: F_3,116_ = 37.37, p < 0.0001), *M. euphorbiae* (nymphs: F_3,116_ = 11.98, p < 0.0001; adults: F_3,116_ = 20.67, p < 0.0001), and *M. pseudorosae* (nymphs: F_3,116_ = 32.59, p < 0.0001; adults: F_3,116_ = 48.47, p < 0.0001) compared with the other three lady beetle species, while *P. quatuordecimpunctata* consumed the fewest adults of these three aphid species and the fewest nymphs of *M. persicae* and *M. euphorbiae. C. septempunctata* consumed the lowest numbers of *M. pseudorosae* nymphs compared with the other three beetle species.

Lady beetles generally consumed fewer *M. albifrons* (Figure 1) compared with the other three aphid species. *C. trifasciata* and *P. quatuordecimpunctata* consumed a greater number of *M. albifrons* nymphs compared with the other two beetle species (F3,116 = 11.86, p < 0.0004); *C. trifasciata* also consumed the greatest number of lupine aphid adults (F3,116 = 6.46, p < 0.0006).

### Field observations

All four lady beetle species were found on *S. tuberosum*, while only *H. axyridis* and *P. quatuordecimpunctata* were found on *R. multiflora* and only *C. trifasciata* and *P. quatuordecimpunctata* were found on *L. polyphyllus* (Table 1). There were significant differences in mean numbers of lady beetle species documented in two of the three vegetation types observed. The most abundant species in *S. tuberosum* was *P. quatuordecimpunctata*, followed by *C. septempunctata* (X^2^= 18.17, p < 0.0001), *H. axyridis* (X^2^ = 22.02, p < 0.0001), and *C. trifasciata* (X^2^ = 18.84, p < 0.0001). On *L. polyphyllus, P. quatuordecimpunctata* was more abundant than *C. trifasciata* (X^2^ = 5.52, p = 0.0188). However, there was no difference in the relative abundance of *P. quatuordecimpunctata* and *H. axyridis* on *R. multiflora*. Although mean lady beetle numbers were higher in six out of the eight occasions where aphids were present compared to absent (Table 1), those differences were not significant.

## Discussion

Consumption rates of the four aphid species differed among the four lady beetle species. With the exception of *M. albifrons, H. axyridis* was the most voracious predator, while *P. quatuordecimpunctata* removed the least prey. There may be a number of reasons for these differences. First, consumption rates may have been affected by the size of the beetles and/or the size of the prey. *C. septempunctata* is the largest of the lady beetles studied, followed by *H. axyridis, C. trifasciata*, and *P. quatuordecimpunctata* (Finlayson unpublished data). Being the smallest in size, *P. quatuordecimpunctata* may be satiated with fewer aphids compared with the other species. *M. albifrons* is larger than the other aphid species, thus fewer *M. albifrons* may satiate a beetle compared with the other species offered. Consumption rate may also be affected by differences in handling (Pervez and Omkar 2005), nutritional suitability of prey (Houck 1991; Roger et al. 2001; Gagné et al. 2002), or chemical deterrence (Pasteels et al. 1983; Nishida and Fukami 1989).

Observations of adult beetles on field plots were generally consistent with expectations based on their consumption of aphids in the laboratory. *H. axyridis* consumed the most *M. pseudorosae* in laboratory trials and was one of two species found in the field with *M. pseudorosae*. *C. trifasciata* consumed the most *M. albifrons* in laboratory trials and was one of two species found in the field with *M. albifrons*. The other beetle species found with *M. pseudorosae* and *M. albifrons* was *P. quatuordecimpunctata*, the species that consumed the second largest number of *M. pseudorosae* and *M. albifrons*, although this difference was only statistically significant for *M. albifrons* nymphs. It is also not surprising to find *P. quatuordecimpunctata* in all observations because this species is probably the most abundant lady beetle in Maine (Finlayson et al. 2008).

**Table 1.**
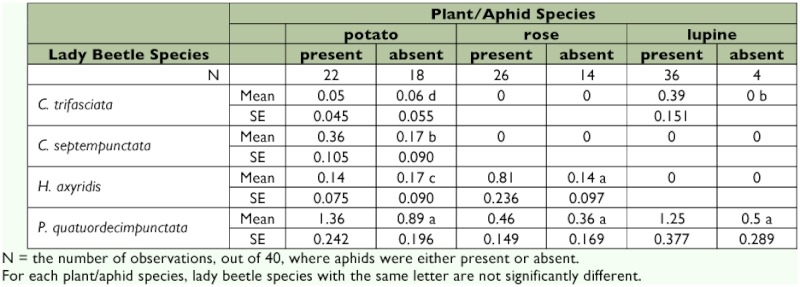
Mean (± standard error) number of lady beetles documented during field observations. Mean beetles documented where aphids were present on vegetation are presented alongside mean beetles that were documented where aphids were absent.

Three of the species tested in this study, *H. axyridis*, *C. septempunctata*, and *P. quatuordecimpunctata*, are not native to Maine (Gordon 1985). Because lady beetle species differ in their prey consumption, decreases in the relative abundance of native species following the establishment of non-native lady beetle species that has been reported in a number of studies (Elliot et al. 1996; Brown and Miller 1998; Colunga-Garcia and Gage 1998; Michaud 2002; Brown 2003; Turnock et al. 2003; Alyokhin and Sewell 2004) may favor some aphid species over others. For example, Alyokhin et al. (2005) observed a significant reduction in both density and the amplitude of annual oscillations of populations of *M. persicae* and *Aphis nasturtii* following the establishment of *H. axyridis* and *P. quatuordecimpunctata*.

When compared with other aphidophagous coccinellid species, *H. axyridis* has been shown to have superior competitive abilities regarding its feeding rate (Michaud 2002), intraguild predation (Hironori and Katsuhiro 1997; Yasuda et al. 2001; Yasuda et al. 2004), and interactions with natural enemies (Dutcher et al. 1999; Saito and Bjørnson 2006; Finlayson et al. 2009). Similarly, in this study, *H. axyridis* exhibited greater prey consumption of three of the four aphid species tested compared with the other lady beetle species tested. The true voracity of *H. axyridis*, however, may have been underestimated because it consumed close to the upper limit of what was made available in trials. Providing more than ten aphids may have improved the resolution of species differences.

*M. albifrons* is native to the study area (Stroyan 1981) and is known to sequester toxic compounds from its host plant that have been shown to cause a “narcotizing effect” on *C. septempunctata* (Gruppe and Roemer 1988). It is thus notable that *H. axyridis* and *C. septempunctata*, both introduced species without historical exposure to *M. albifrons*, consumed the lowest numbers of this species. In contrast, *C. trifasciata*, which is native to the area, consumed the most *M. albifrons* adults. It would seem that *C. trifasciata* may have evolved the ability to tolerate these compounds, whereas the recently introduced non-native species have yet to do so. By virtue of being able to exploit lupine aphids, *C. trifasciata* may enjoy a refuge from prey competition with the non-native species. These differences in prey consumption suggest that different lady beetle species should not be considered equal consumers of aphids.
